# The oculocardiac reflex and depth of anesthesia measured by brain wave

**DOI:** 10.1186/s12871-019-0712-z

**Published:** 2019-03-14

**Authors:** Robert W. Arnold, Aleah N. Bond, Melissa McCall, Leif Lunoe

**Affiliations:** 1 Alaska Children’s EYE & Strabismus, 3500 Latouche St #280, Anchorage, Alaska 99508 USA; 2 Providence Anesthesia Group, Anchorage, Alaska USA

**Keywords:** Trigeminovagal reflex, Trigemino cardiac reflex, Bradycardia, Children, Squint

## Abstract

**Background:**

The oculocardiac reflex (OCR), bradycardia that occurs during strabismus surgery is a type of trigemino-cardiac reflex (TCR) is blocked by anticholinergics and enhanced by opioids and dexmedetomidine. Two recent studies suggest that deeper inhalational anesthesia monitored by BIS protects against OCR; we wondered if our data correlated similarly.

**Methods:**

In an ongoing, prospective study of OCR/TCR elicited by 10-s, 200 g square-wave traction on extraocular muscles (EOM) from 2009 to 2013, anesthetic depth was estimated in cohorts using either BIS or Narcotrend monitors. The depth of anesthesia was deliberately varied between first and second EOM tested.

**Results:**

From 1992 through 2013, 2833 cases of OCR during strabismus surgery were monitored. Excluding re-operations and cases with anticholinergic, OCR from first EOM traction averaged − 20.2 ± 21.8% (S.D.) with a range from − 95 to + 25% in patients aged 0.2 to 90 (median 6.5) years. We did not find correlation between %OCR and brain wave for 97 patients with BIS monitoring and 91 with Narcotrend. With intra-patient controls between first and second muscle, the difference in brain wave did not correlate with difference in %OCR for BIS (r = 0.0002, 95% C. I -0.0002, 0.002, *p* = 0.30) or for Narcotrend (*r* = − 0.001, 95% C. I -0.004, 0.001, *p* = 0.32). Secondary multi-variable analysis demonstrated significant association on %OCR particularly with BIS monitor, opioid, propofol and nitrous oxide concentration in the second EOM tensioned. Sevoflurane concentration correlated better with BIS monitor in second and third EOM tension. %OCR correlated with younger age (*p* < 0.01). OCR with rapid onset was more profound than those with gradual onset (difference in means 18, 95% C. I 10, 26%).

**Conclusions:**

We were unable to confirm a direct correlation between brain wave monitor and OCR when using multifactorial anesthetic agents. The discrepency with other studies probably reflects direct impact of inhalational agent concentration and less deliberate quantification of EOM tension. We found no level of BIS or Entropy EEG monitoring that uniformly prevents OCR.

**Trial registry:**

NCT03663413.

Data: http://www.abcd-vision.org/OCR/OCR%20Brainwave%20de-identified.pdf.

## Background

The oculocardiac reflex (OCR) is a trigemino-vagal reflex associated with manipulations of the eye and orbit and specifically tension on the extraocular muscle during strabismus surgery [1]. The term Trigemino Cardiac Reflex (TCR) has efferent cardiac, respiratory and gastric vagal influence stimulated by any branch of the fifth cranial nerve [2, 3]. Of TCR and other vagal reflexes including diving response, the oculocardiac is not usually the most profound, however it has great intersubject variability occasionally producing profound bradydysrhythmias [1].

Anesthesiologists and ophthalmic surgeons make attempts to protect patients from the occasional profound oculocardiac reflex [4]. Gentle tension and release of tension by the ophthalmic surgeon limit or reverse the bradycardia [5, 6]. OCR can be inhibited by retrobulbar block and with anticholinergic medications [7], particularly when administered intravenous or intraglossally [8]. OCR is augmented by some opioids [9–11] and by dexmedetomidine [12, 13].

Yi, et all in 2008 reported that BIS values less than 50 in children undergoing strabismus surgery under sevoflurane anesthesia had less OCR [14]. Karaman, et al. in 2016 reported their experience suggesting that their cohort with lower BIS scores with nitrous oxide / desflurane general endotracheal anesthesia were associated with less bradycardia during deliberate, but non-quantified, extraocular muscle traction [15]. Was the reduction in OCR in these studies due to the inhalational agent concentration or the BIS score? Both anesthesia and surgeon have an influence on OCR. Depth of anesthesia is impacted by several components including inhalational agent concentration, nitrous oxide, opioids, propofol, sedatives, etc. OCR is also markedly influenced by the duration, form and amount of tension [5, 6, 16] on different extraocular muscles that were not controlled in former papers. We hypothesized that, over a range of anesthesia depths monitored by two different brain wave devices, we would find a correlation between specifically controlled oculocardiac reflex and lighter anesthesia.

## Methods

**Fig. 1:**
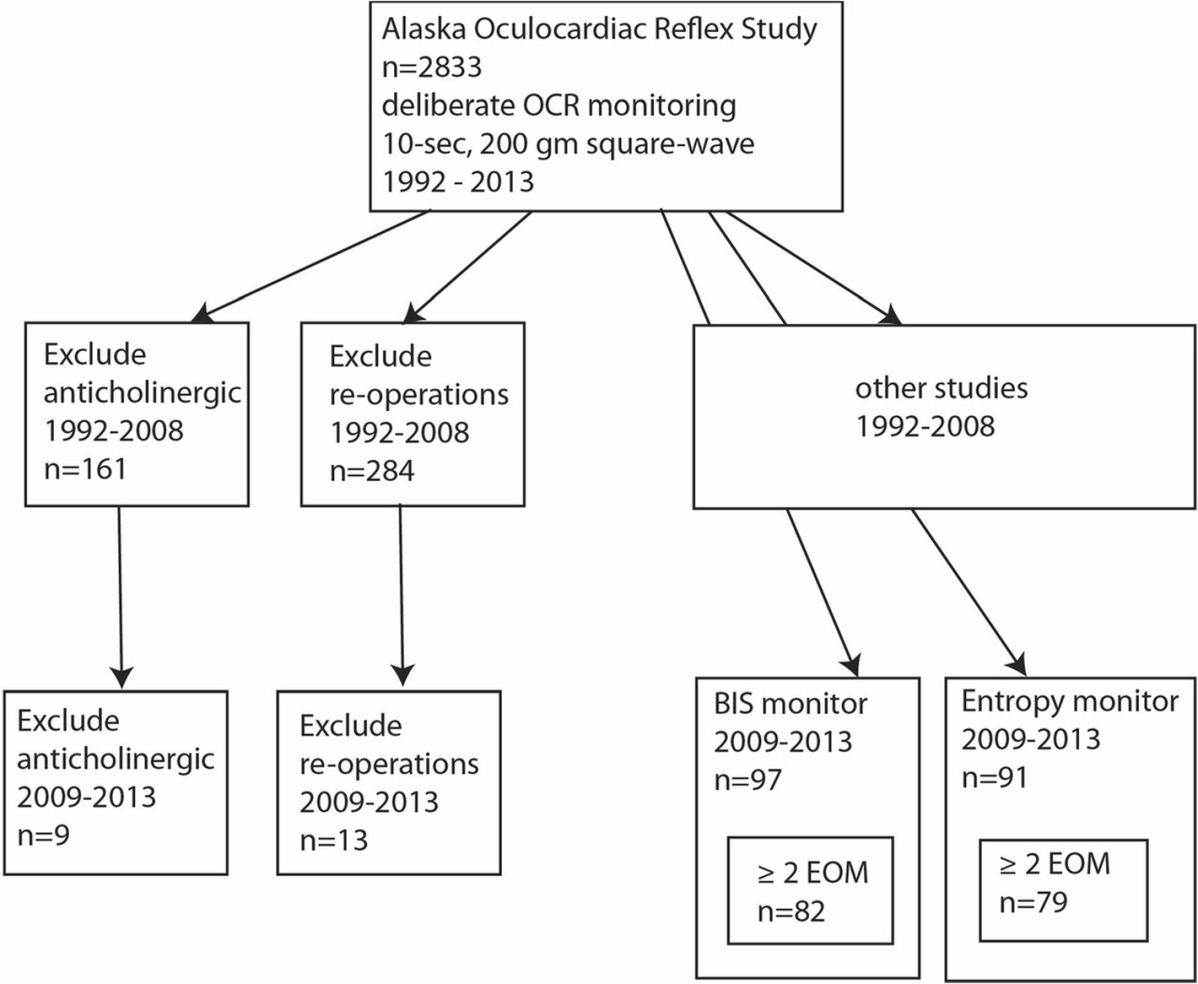
Break down of patients recruited in the ongoing Alaska Oculocardiac Reflex Study. From 2009 through 2013, sequential patients in two different outpatient surgery centers were monitored with either BIS or Narcotrend brain wave monitors. Patients initially receiving anticholinergic medication or patients returning for re-operations were excluded

From 1992 through 2013, patients undergoing strabismus or orbital surgery had deliberate, quantified extraocular muscle tension with prospective monitoring of electrocardiograph and anesthetic variables in an ongoing, HIPAA-compliant study with institutional review board approval from Providence Hospital (ClincalTrials.gov NCT03663413). This ongoing observational study complies with the Declaration of Helsinki and utilizes de-identified data; written informed consent has not been a requirement of the study. The intent of the study has been to monitor the oculocardiac reflex in community surgery centers with choice of agents up to the anesthesia teams. Since profound OCR is a relatively rare event, our ongoing prospective study using identical extraocular muscle stimulation provides a unique opportunity to observe large numbers of patients over time. This present report covers prospectively recorded anesthetic parameters in two cohorts representing different surgery centers and brain wave monitors, but identical strabismus rectus muscle stimulation to elicit oculocardiac reflex (Fig. 1). Patients were selected from all consecutive strabismus surgery patients of one surgeon (RWA) including ASA 1 and 2 patients some with developmental disabilities. There was no health or age related bias directing patients to either surgery center, however younger patients were more likely to receive pre-operative sedation- that may serve as a confounder with respect to age. Anesthesia staff was equally likely to increase or decrease anesthesia depth over the course off the surgery. The known confounder dexmedetomidine was not used in this study group [13]. Excluded were any patients given anticholinergic medication before OCR was measured and re-operations.

At the choice of the outpatient anesthesia team, some patients were given premedication: oral midazolam 0.5 mg/Kg. During induction, some patients were given IV fentanyl 1–2 mcg//Kg and/or propofol 3 mg/Kg. The children were induced with inhaled N2O and sevoflurane and then intravenous catheter inserted. No pre-operative, or induction anticholinergic was given. Most patients had laryngeal mask airway however some had general enotracheal. Few required rocuronium 0.5 mg/Kg. The anesthesiologist deliberately varied anesthetic agents, and specifically the inhalational agent concentration within customary levels to allow observation of the impact of lighter and deeper levels of anesthesia on the same patient.

From 2009 through 2013, each patient was monitored for electrocardiograph, end-tidal agent and CO2 concentration, and brain wave. Anesthetic depth was estimated using bispectral index (BIS) monitors (BIS XP, Aspect Medical Systems, Newton, Mass) in one center, and Narcotrend monitors (MonitorTechnik, Bad Bramstedt, Germany) in another surgical center. The monitors were applied to the forehead and temple according to manufacturer’s instructions, and covered with adhesive, transluscent plastic drape after iodophore preparation. Additional monitoring included end-tidal agent concentration, CO2 monitoring and electrocardiograph. Oculocardiac reflex (OCR) was monitored during deliberate, 10-s, square wave tension on carefully isolated extraocular rectus muscles. Some cases involved surgery on just one extraocular muscle, while the majority of cases involved surgery on more than one muscle with time for heart rate to return to stable baseline between deliberate tension.

Oculocardiac reflex is reported as the maximum percent change in heart rate from stable, pre-tension ECG to the maximally different heart rate induced by the 10-s, deliberate extraocular muscle tension. Stability in brain wave monitor also preceded OCR monitoring. Although some define OCR as a 20% drop in heart rate [17], percent change better allows for comparison between children and adults with different resting heart rates. A negative percent represents bradycardia ((OCR heart rate – pre-heart rate)/pre-heart rate), whereas a positive percent oculocaridac could represent tachycardia. By this definition, more OCR (greater minus percent) implies more bradycardia and lower heart rate. We differentiated between oculocardiac with gradual onset from those with rapid onset- defined by at least 25% heart rate change in the first two seconds after extraocular muscle tension. For practical approach reasons, for uniformity, and because it produces the greatest OCR of the recti [18], inferior rectus muscles were most commonly tested. An example of rapid-onset, inferior rectus oculocardiac reflex can be observed at https://vimeo.com/robertarnold/oculocardiacreflex.

### Statistical methods

Continuous data %OCR and brain wave monitor readings were analyzed with linear regression with calculations for coefficient of regression with 95% confidence intervals and Pearson product moment correlation with *p* values before and after adjustment for multiple comparisons. For cases with two muscles recorded of a given patient, the difference in %OCR from muscle 1 to muscle 2 was correlated to the difference in brain wave monitor reading at time of deliberate muscle tension. Linear regression was also employed comparing %OCR to age.

A multi-variable analysis then sought contributing related explanatory variables as might influence depth of anesthesia (midazolam, propofol, opioid, nitrous oxide, inhalational agent concentration). A *p* value less than 0.05 was considered significant. To address interaction, interaction terms were generated between pairs of explanatory variables with or without Robust standard errors and none of these contributed to additional significance.

A student’s t-Test was employed to determine difference in means of cases showing rapid versus gradual onset OCR.

### Sample size

For a correlation to detect an r of 0.3 or − 0.3 given power of 0.8 and alpha of 0.05, sample size would be 85.

For t-Test, given a power 0.8 and alpha 0.05, we estimate that we could detect a difference of 10% from a mean of − 20% OCR given S.D. of 18% with 51 samples.

## Results

From 1992 through 2013, 2833 consecutive cases of OCR during strabismus surgery were monitored. Excluding 297 re-operations and 170 cases with anticholinergic, OCR from first EOM traction averaged − 20.2% (± 21.8% S.D., 95% C.I. -81, + 5%) with a range from − 95 to + 25% in patients aged 0.2 to 90.2 (median 6.5) years. No adverse cardiac event requiring rescusitation occured despite asystole occasionally longer then ten seconds. Release of tension was employed as the primary therapy for profound oculocardiac reflex. Some anethesiologists then employed intravenous anticholinergic before extraocular muscle manipulation resumed.

The number of patients with EEG monitoring (BIS 97 and Narcotrend 91) during strabismus surgery was 188 with a breakdown of their characteristics given in Table 1. Their initial OCR was − 19 ± 18% and the proportion of our patients with initial OCR greater than 10% was 56% while the proportion greater than 20% bradycardia was 43%. 98% of EOM traction was on the inferior rectus with 2% on the superior rectus. Table 2 shows the Pearson Product-moment correlation between %OCR and brain wave monitoring for BIS and Narcotrend for first, second and third EOM traction. The *p*-value is presented and adjusted for multiple comparisons. We did not detect a significant correlation under any of these conditions.

**Table 1:** Demographics of cohorts

	BIS	Narcotrend
*n*	**97**	**91**
male/female	48/49	41/50
age minimum	1.3	0.5
age maximum	77	73
age mean	18 ± 23	17 ± 20
age median	5.7	6.5
Neuro disorder	5%	10%
opioid	52%	25%
rapid onset	16%	5%
midazolam	20%	5%
one EOM	9%	22%

Opioid and midazolam are the percent of patients receiving these drugs as a part of their anesthesia. Rapid onset are the percent pf patients with more than 25% drop in hear rate in the first two seconds. One EOM are the percent of cases with only one extraocular muscle operated. BIS and Narcotrend are the brain wave monitors used for each cohort

**Table 2:** Primary Outcome Variables: correlations between brainwave monitor (BIS and Narcotrend) and oculocardiac reflex (%OCR) for first, second and third extraocular muscle with 10-s, 200 g tension during strabismus surgery

	n	Pearson	*p*	*p*’
%OCR1				
BIS	97	− 0.12	0.24	0.43
Narcotrend	91	0.018	0.86	0.98
%OCR2				
BIS	82	−0.13	0.23	0.41
Narcotrend	79	−0.12	0.29	0.49
%OCR3				
BIS	54	−0.12	0.40	0.64
Narcotrend	39	0.08	0.62	0.86
∆OCR				
∆BIS	82	−0.12	0.30	0.51
∆Narcotrend	79	−0.11	0.32	0.54

Pearson is the Product-Moment Correlation with p probability and p’ adjusted for multiple comparisons. The lower table shows the correlations between the difference between OCR and monitor values with intra-subject control between first and second extraocular muscle tension in a given case

Table 2 also shows the difference in %OCR between first and second EOM correlated with the difference in brain wave monitoring- our primary outcome variable with intra-subject control to test our hypothesis that deeper anesthesia monitored by brainwave would influence OCR. With deliberate change in agent percent and other anesthetic agents as directed by the anesthesiologist between first and second extraocular muscle tension in multiple-muscle cases, the average absolute difference readings between first and second reading at the time of oculocardiac measurement for BIS was 14.2 ± 16 units and for Entropy 16.3 ± 17 units. The interval between first EOM and second EOM in 134 cases was 16 ± 6 min while the interval between second and third EOM in the 74 cases was 8 ± 4 min. With BIS monitoring and the 82 cases with two-muscle comparison, no correlation between delta BIS and delta %OCR was found (r = 0.0002, 95% C. I -0.0002, 0.002, Pearson R (80) = − 0.12, *p* = 0.30). For the 79 two-muscle strabismus cases monitored with Narcotrend, no correlation between delta Narcotrend and delta %OCR was found (r = − 0.001, 95% C. I -0.004, 0.001, Pearson R (77) = − 0.11, *p* = 0.32).

Table 3 shows multi-variable analysis of potential determinates of %OCR including brain wave monitor, agent concentration, end tidal CO2, and presence of pre-op midazolam, intravenous fentanyl and nitrous oxide for first, second and third EOM. Also, to facilitate comparison with other publications [17], OCR defined as 10% bradycardia and 20% bradycardia are analyzed. The oculocardiac reflex did not correlate with end-tidal carbon dioxide levels. No consistent pattern of interaction was noted except for second EOM and BIS monitor. Even then, for the 82 strabismus cases with %OCR2 correlated against BIS monitor, minimal correlation was noted (*r* = − 0.002, 95% C.I. -0.006, 0.001, Pearson r (80) = − 0.133).

**Table 3:**
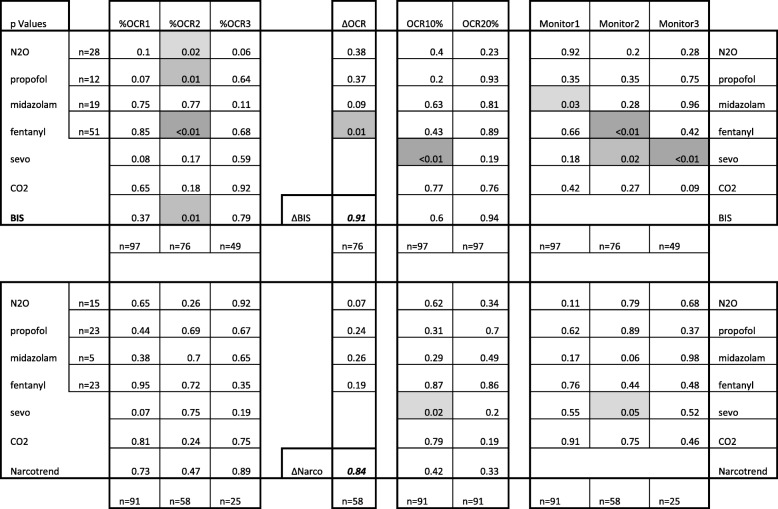
Multi-variable linear regression Model

The significance (*p* value) for various anesthetic paramaters on oculocardiac reflex utilizing the brain wave monitors BIS (top seven rows) and Narcotrend (bottom seven rows.) First three columns represent separate analyses for percent oculocardiac reflex with the first extraocular muscle (EOM; 98% inferior rectus), the second EOM and the third EOM in strabismus surgery cases. Next column ∆OCR represents the difference between %OCR1 and %OCR2 correlated with the difference in brain wave monitors (∆BIS and ∆Narcotrend). Next two colums added for comparison with other authors who employ ordinally defined oculocardiac reflex OCR10% and OCR20% as proportion with 10% bradycardia or 20% bradycardia. Final three columns determine impact of anesthetic variables on the readings from the BIS and Narcotrend monitors for first EOM, second EOM and third EOM observations. Far right and left columns highlight covariates nitrous oxide concentration (N2O), induction propofol, premedication oral midazolam, induction fentanyl, endtidal sevoflurane concentration (sevo), end-tidal carbon dioxide (CO2) and brain wave monitor reading. Below each column in the number of patients in that cohort

**Fig. 2:**
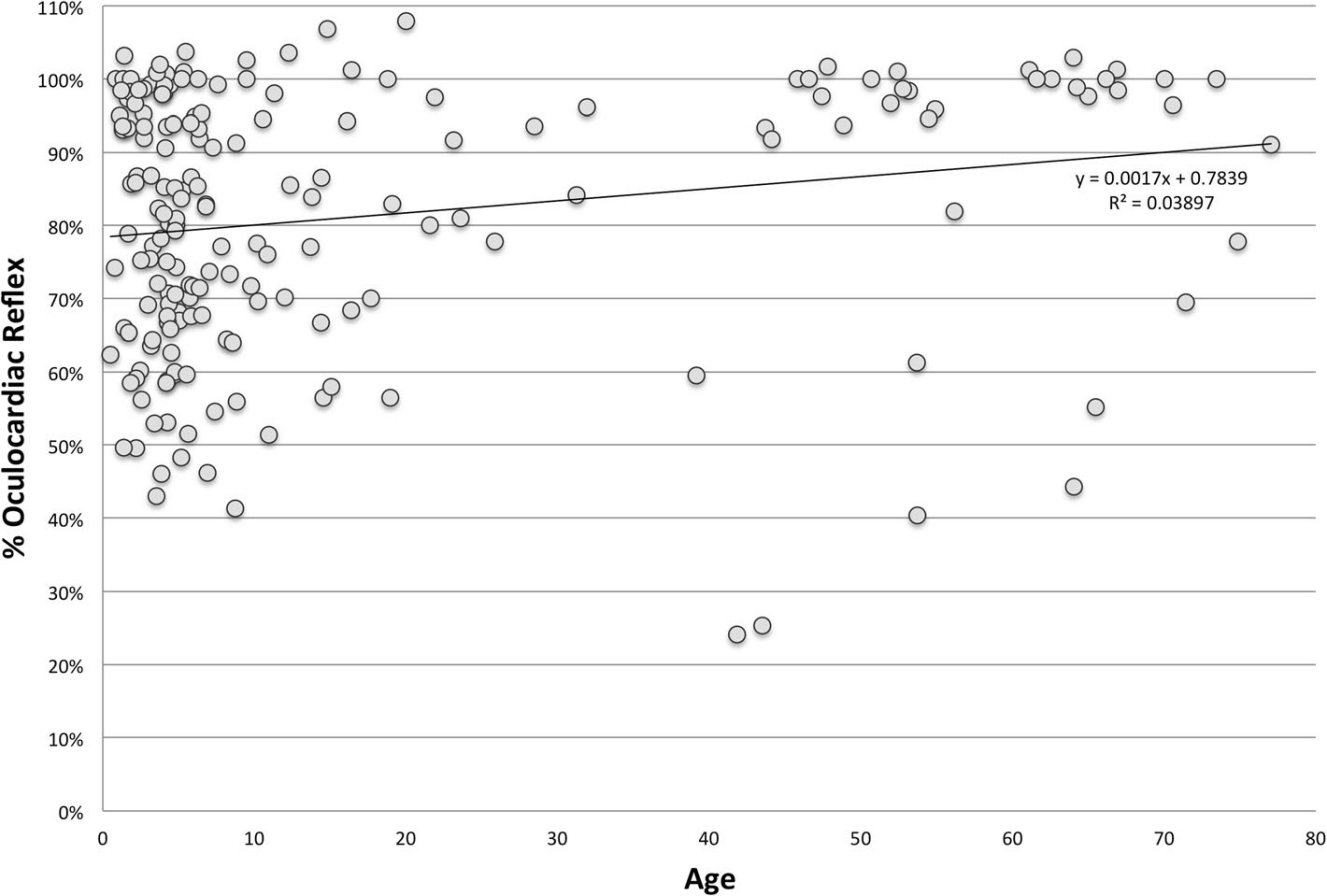
Influence of age on percent oculocardiac reflex. *N* = 188

For this study group, we found that %OCR correlated with younger age (Fig. 2; *r* = 0.002, 95% C.I. 0.0005, 0.003, Pearson r (186) = 0.197, *p* < 0.01.) The linear regression formula is %OCR = 0.17 (age) -21.6%. OCR with rapid onset (*n* = 21, − 33 ± 18%) was more profound than in those with gradual onset (− 16 ± 18%; t-Test p < 0.01, difference in means 18, 95% C.I. 10, 26%).

## Discussion

We deliberately monitored uniformly-generated oculocardiac reflex with brain wave monitoring in community surgery centers, then adjusted anesthetic parameters to allow patients to be internal controls for change in depth of anesthesia with two types of brain wave monitors. The 10-s, 200 g square-wave tension that generated the OCR for these 188 patients (− 19 ± 18%) which closely resembled the − 20 ± 22% from more than 2 decades of similar data collection. We employed continuous variables to increase the power of our observations. We were unable to find a significant correlation between OCR and brain wave monitor levels alone, and we are not sure whether our observations of lack of brain wave correlation would impact other forms of trigemino cardiac reflex (TCR). This differs from the two other direct investigations on this subject and a systematic review of OCR/TCR that controlled other anesthetic conditions varying only inhalational agent concentration within pre-defined BIS monitor readings.

In the first publication on OCR and BIS, Yi, et al. [14] had three cohorts of 28 children varying sevoflurane to achieve BIS levels of 40–49, 50–59 and 60–69. They employed pre-op IM atropine and thiopentone in all patients. The surgeon was not masked to BIS and agent concentration and did not quatify EOM tension or duration, however tension was apparently gentle such that their initial ordinal definition of OCR had to be switched from 20% down to only 10% bradycardia. They reported more lateral rectus muscles tensioned than medial rectus and stopped traction if their defined 10% OCR was observed. The incidence of 10% OCR was 11% for deep cohort, 32% for mid cohort and 71% for the light anesthesia cohort.

In the other recent study on BIS and OCR, Karaman et al. also found less oculocardiac reflex in a study arm with lower BIS [15] Their prospective study used general endotracheal anesthesia with fentanyl, no anticholinergic and maintenance with 50% nitrous oxide and desflurane so the deep group of 31 children had BIS < 50 while the light group of 28 children had BIS ≥50. OCR, ordinally defined as 20% heart rate drop or arrythmia was 25% in the deep group, but 64% in the light group. Extraocular tension was deliberate but not quantified and a difference between medial rectus and lateral rectus was only noted in the light anesthesia group.

A systematic review of Trigemino-Cardiac Reflex (TCR) found a weak 1.2 fold increase with lighter anesthesia, but a 4.5 fold pooled risk of asystole when light anesthesia is compared to deeper anesthesia [19].

As a secondary outcome, we employed multi-variable analysis with respect to the continuous variable %OCR, additional anesthetic agents had a significant effect, noted more with BIS monitoring than with Narcotrend and more so with the second EOM than the initial EOM. This may be due to residual impact of midazolam pre-medication and the induction agents fentanyl and propofol. Compared to the studies by Yi [14] and Karaman [15], we had several additional pharmacologic confounders than just varied inhalational sevoflurane or desflurane. We suspect the levels of those agents, more so than the actual BIS reading itself, can impact OCR defined as a small percent change in heart rate, when the surgeon’s extraocular tension is not uniformly controled. The amount and duration of EOM tension have a major impact on OCR [5, 6].

Both BIS and Narcotrend measure EEG activity, but employ different algorithms to derive a value [20]. Similar to others [21], we noted differences in the ability of Narcotrend compared with BIS to detect correlations that impacted OCR. Though our study was not intended to determine superiority of one brain wave monitor over the other, we did not have uniform correlation between brain wave and sevo concentraion but observed slightly better correlation between BIS and agent concentration than with Narcotrend (Table 2).

Under our current anesthetic conditions, the percent oculocardiac reflex correlated with age such that younger patients had a slightly greater heart rate change. This differs from our earlier findings also reported as percent OCR [22], perhaps due to different anesthetics and/or to pre-operative sedation that was more often employed in younger patients [5]. In prior studies, halothane and flurane were used with nitrous oxide, whereas most current cases employ sevoflurane. Midazolam, fentanyl or other agents (dexmedetomidine) are currently used pre-operatively mainly in children. In our experience, opioids and dexmedetomidine augment oculocardiac reflex; the latter was not employed in this study group [12]. We do not know whether opioids and dexmedetomidine augment other forms of TCR.

Approximately 11% of our cases are characterized by rapid onset- and these cases have substantially greater oculocardiac reflex.

Strengths of our study are the wide inclusion of strabismus patients from young children to adults, the strict control of extraocular muscle stimulation primarily on the inferior rectus muscles and our use of continuous variables for oculocardiac reflex and brain wave monitor readings. To enhance comparability with other historic studies, we also provide out data as if OCR was defined ordinally as 10% bradycardia, or 20% bradycardia. Given this, and the variety of anesthetic agents employed in community surgery centers, our study is genalizeable to a wide variety of patients. Weakness of our study include a non-uniform anesthetic protocol and more pre-operative sedation in younger patients; ideal conditions might involve double-blind randomization of the variable amount of one anesthetic component such as inhalational agent concentration unbeknown to anesthesiologist and to strabismus surgeon. We had sufficient numbers to have demonstrated a substantial correlation had there been one, but halved the cohort numbers employing two different types of monitors not intending to determine inferiority of one against the other.

## Conclusions

Our observations, especially with BIS brain wave monitor, provide some more evidence that deeper anesthesia can provide some protection against the oculocardiac reflex. Further study is needed to fully delineate the relative roles of various anesthetic agents. Due to the high inter-subject variability of oculocardiac reflex, and our case-by-case use of a variety anesthetic agents, we observed some cases of profound oculocardiac reflex despite BIS and Narcotrend monitor readings indicating deep anesthesia.

## Abbreviations

ASA: Amercian Society of Anesthesiologists
BIS: Bispectal index
C.I.: Confidence interval
CO2: Carbon dioxide
ECG: Electrocardiograph
EEG: Electroencephalogram
EOM: Extraocular muscle
HIPAA: Health Insurance portability and accountability” act”
IRB: Institutional review board
N2O: Nitrous oxide
OCR: Oculocardic reflex
S.D.: Standard deviation
TCR: Trigemino Cardiac Reflex
